# Experimental studies on the mechanical properties of garlic scape

**DOI:** 10.1371/journal.pone.0344722

**Published:** 2026-04-16

**Authors:** Jing Yang, Baogang Xian, Shengben Lin, Xin Guo

**Affiliations:** College of Electron-mechanics and Automobile Engineering, Tianshui Normal University, Tianshui, Gansu, China; National Chung Cheng University, Taiwan & Australian Center for Sustainable Development Research and Innovation (ACSDRI), AUSTRALIA

## Abstract

Garlic plants are composite materials with multi-scale structures, and the efficient separation of garlic scapes from pseudo-stems is the core challenge in the mechanized harvesting of garlic scape. To deeply elucidate the separation mechanism of garlic scapes and pseudo-stems, this study first confirmed the orthogonal anisotropy characteristics of garlic plants through microscopic morphology characterization. Based on the theory of composite material mechanics, a mechanical model of garlic plants was constructed. Tensile, compressive, and shear tests were conducted on intact garlic plants and their respective components (garlic scapes and pseudo-stems) using a universal testing machine, and key mechanical parameters were obtained systematically. Significant differences were identified between garlic scapes and pseudo-stems in terms of elastic modulus, peak strain, tensile strength, and shear strength through significance analysis, providing a mechanical basis for their selective separation. To verify the reliability of the experimental data, finite element simulation was performed to validate compression tests of garlic plants. The results indicated a high degree of agreement between the experimental and simulated curves, confirming the validity of the compressive tests. Furthermore, theoretical verification of the shear test results was carried out based on the mixing rule of composite materials, which revealed that the theoretical values of the plant elastic modulus were higher than the measured values in the tests. Combined with scanning electron microscopy (SEM) analysis, it was shown that this discrepancy primarily stemmed from the uneven slippage occurring inside the garlic scapes during the shearing process. This study systematically clarifies the differences in mechanical properties and the underlying separation mechanism between garlic scapes and pseudo-stems, providing critical parameters and theoretical foundations for the efficient and low-damage separation of garlic scapes from pseudo-stems in mechanized harvesting.

## 1. Introduction

Garlic scape is the flower stems of garlics [1], which is widely cultivated in various regions of China. Due to high nutritional value, it is the vegetable with the largest reserves and the longest storage period in China’s cold storage industry. Garlic scape harvesting has certain particularity. The exterior of garlic scape is wrapped with a certain thickness of pseudo-stems. The essence of mechanized harvesting garlic scape is the precise separation of garlic scapes from pseudo-stems, ensuring “garlic scape breaking without pseudo-stem damage” [2,3]. This approach facilitates the efficient harvesting of garlic scapes while preserving the structural integrity of pseudo-stems, thereby ensuring the subsequent growth of garlic. Currently, “the method of clamping garlic scape” is commonly adopted in practical production to achieve this selective harvesting objective. During the separation process, garlic scapes are prone to mechanical damage caused by compression, tensile, shear, and so on. This not only impacts the quality of frozen storage and edibility but also damages the structure of pseudo-stems, thereby affecting the subsequent growth of garlic. Therefore, studying the biomechanical properties of garlic plants and their components, and comprehensively understanding the separation mechanism between garlic scapes and pseudo-stems are crucial for optimizing mechanized harvesting techniques and improving storage quality.

In recent years, research on the mechanical properties of garlic scapes has been conducted by some scholars. Yuan et al. [4] performed tensile tests on three distinct garlic scape varieties, revealing that the tensile strength of garlic scapes is influenced not only by the variety of garlics but also by their moisture content: the lower the moisture content, the higher the tensile strength. Zhu et al. [5] studied the mechanical properties of the Cangshanpuke garlic variety. They divided a garlic scape into five segments from the top to the root, and successively conducted tensile and compressive tests on them. The tensile test results showed that the tensile stress borne by the garlic scape decreased sequentially from the top to the root. Similarly, the compression test results demonstrated that the radial compressive deformation of the garlic scape also exhibited a gradual decreasing trend from the top to the root. The ideal clamping position for a robotic end-effector is at the top segment of the garlic scape. Geng et al. [6,7] selected the garlic scape variety Xu918 and performed compression tests using a texture analyzer. The study found that the maximum radial compressive stress that the clamped area can withstand is 0.81 MPa, providing a theoretical reference for ensuring secure clamping without damaging garlic scapes. These findings indicate a valuable reference for the theoretical analysis of the separation between garlic scape and garlic plant. Wang et al. [8,9] conducted an analysis of force during the bolting process and established the correlation between bolting force and time. Previous studies on the mechanical properties of garlic scape have mostly focused on segmented tests of their different parts and quantitative analysis of mechanical properties during the bolting process. However, from the perspectives of composite material mechanics, research that takes the entire garlic plant as the research object, systematically compares and analyzes the differences in mechanical properties between pseudo-stems and garlic scapes under multiple loading modes, and further elucidates the mechanical mechanism of “garlic scape breaking without pseudo-stem damage” during mechanized harvesting remains rarely reported. The mechanized harvesting of garlic scape is essentially a selective separation process. Without a systematic analysis of the mechanical properties of entire plants and their components, it is difficult to fundamentally reveal the mechanism of the scape-stem separation. Therefore, this study aims to fill this gap. By systematically analyzing the mechanical behavior of garlic scapes and pseudo-stems, it provides theoretical basis and design guidance for high-efficiency and low-damage mechanical harvesting of garlic scape.

At present, numerical simulation has become a necessary and powerful tool to settle the problems of modern engineering [10]. Ashtiani MS et al. [11] employed the finite element method to simulate the mechanical damage of grapefruit subjected to external compressive forces. Pu et al. [12] experimentally analyzed the stress distribution in the peel and flesh of navel oranges under different compression scales, and validated the accuracy of the quasi‑static compression tests through finite element simulation. Cheng et al. [13] simulated and analyzed the compression and fracture processes of cerasus humilis fruit. The results showed that the force-displacement curves were in good agreement with the experimental data, and the fruit cracking process was consistent with the experimental observations. Ademola M. Aina et al. [14] used Finite Element Analysis (FEA) to simulate the mechanical response of papaya under compressive loading, determining the optimal single material model and investigating the variations in mechanical response when loaded along two different directions.

The finite element method has been successfully applied in the simulation analysis of fruits and other crops under external loads [10–19]. However, research on FEM simulation of the mechanical response of garlic plants under compressive loads remains scarce.

In this study, a combined method of experimentation and numerical simulation was employed to investigate the mechanical properties of garlic plants and their components. This research provides data support and a theoretical basis for in-depth clarification of the mechanism of the mechanical harvesting of garlic scape. This study has three contributions:

(1) Through SEM (scanning electron microscopy) images analysis, the orthogonal anisotropy characteristics of garlic plants were determined. Using a universal testing machine, tensile, compression, and shear tests were conducted on intact garlic plants, garlic scapes, and pseudo-stems, respectively, obtaining key parameters including elastic modulus, ultimate strength, and peak strain.
(2) Based on the theory of composite material mechanics, the complete mechanical parameters of garlic plants and their components were determined. Numerical simulation method was employed to validate the obtained mechanical parameters. Specifically, finite element simulation was used to verify the rationality and effectiveness of the compression tests of garlic plants. Furthermore, the shear tests were validated and analyzed by applying the mixing rule of composite materials, and an in-depth analysis of the inherent mechanisms underlying the shear test results was conducted from a microstructural perspective.
(3) Through systematic analysis and comparison of the mechanical test results of garlic scapes and pseudo-stems, the separation mechanism between garlic scapes and pseudo-stems during mechanical harvesting was revealed, which provides a theoretical basis for optimizing the key parameters of harvesting equipment.

## 2. Materials and methods

### 2.1. Test materials

The test materials of the white garlic variety used in the experiments were purchased from local farmers at the Sanyangchuan Garlic Planting Base, Tianshui City, Gansu Province, China in May 2025. A total of 80 plants were randomly selected. Among them, 64 intact and undamaged samples were chosen for mechanical property tests, and the remaining of them were reserved as backups. The garlic plant diameter (measured at 80–100 mm above the ground), the average diameter of garlic scape (calculated as the mean of the root, middle, and top section diameters), and the pseudo-stem thickness were all repeatedly measured 20 times using a digital vernier caliper with an accuracy of 0.01 mm.

### 2.2. Test equipment

The tests site was selected in the Mechanics Laboratory of the School of Electrical and Automotive Engineering of Tianshui Normal University. Tensile, shear, and compression tests were performed using a Universal Testing Machine (10000 N capacity; 0.18% accuracy; Wance Company, Shenzhen, China) equipped with corresponding fixtures. The force-deformation relationship can be automatically recorded by a computer in a point-by-point manner, and the coordinates and structural parameters of each point can be read by specified files [19]. The tests were implemented according to the standard of GB/T1040.5-2006 Tensile Methods for Plastic and Composite Materials (China).

To elucidate the correlation between microstructural characteristics and mechanical properties of garlic scapes, microstructural observations were conducted using a scanning electron microscope (Hitachi Reguius 8100, Tokyo, Japan). The samples were dried using a critical point dryer (Quorum K850, UK). To prevent the samples from being charged during the SEM observation, the surface of samples was coated with Pt using a sputter coater (Hitachi MC1000, Japan).

### 2.3. Moisture content and density

In this study, the oven-drying method was employed to determine the moisture content. Six intact garlic plants were randomly selected, and the garlic scapes were extracted from the garlic plants and weighed. The samples were placed in an oven (DG450B, Guangzhou Degong Machinery Equipment Co., Ltd., China, 5–250°C temperature range) and dried at 105°C for 8 hours [20–21], after which they were weighed again. The moisture content was then calculated using formula (1).


$$Y=\frac{M_{\text{1}}-M_{\text{2}}}{M_{\text{1}}}\times \text{1}\text{0}\text{0}\%$$ (1)

where *Y* is the moisture content of garlic scape (%); *M₁* is the weight of garlic scape before oven-drying (g); and *M₂* is the weight of garlic scape after oven-drying (g).

Density was determined using the drainage method. Specific procedures were as follows: Pseudo-stems and garlic scapes were separately peeled from garlic plants, cut into uniform segments with scissors, and accurately weighed on an electronic balance (Shanghai Puchun Metrology Instruments Co., Ltd., China, ± 0.01g accuracy). Using purified water as the measuring medium, the initial water level in the graduated cylinder was recorded. The samples were then placed into the cylinder, and the final water level was recorded. Subsequently, the density was calculated using formula (2). To ensure data reliability, each measurement was repeated three times, and the experimental data were expressed as the mean value of three measurements for subsequent statistical analysis.


$$\rho =\frac{m}{{V-V}_{\text{0}}}\times \text{1}\text{0}\text{0}\text{0}$$ (2)

Where *ρ* is the density of garlic scapes or pseudo-stems (kg/m^3^); *m* is the mass of garlic scapes or pseudo-stems (g). *V* is the submerged volume of water (ml). *V*_*0*_ is the initial volume of water (ml);

### 2.4. Establishment of the geometrical model

**Fig 1:**
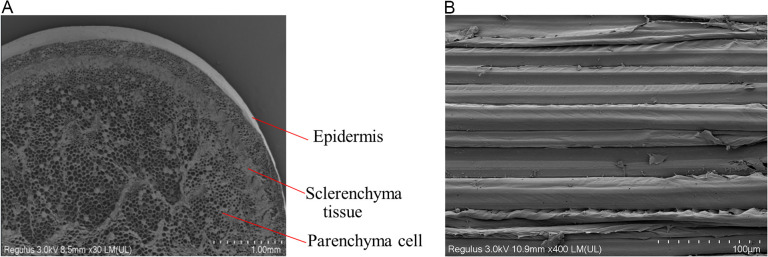
SEM images of garlic scapes. **(a)** Transverse-sectional microstructure of garlic scape. **(b)** Longitudinal -sectional microstructure of garlic scape.

To develop a more accurate mechanical model, the structure and tissues of garlic scapes were first investigated. Fig 1 showed the SEM images of a garlic scape. The garlic scape structure consists of the epidermis, sclerenchyma cells and parenchyma cells. In the transverse section, the garlic scape exhibits a circular profile with sclerenchyma tissues distributed in an annular pattern, enclosing vascular bundles and parenchyma cells in the inner region. In the longitudinal section, the cellular tissues and vascular bundles are observed to exhibit a continuous, ordered, and elongated morphology, which runs parallel to the growth direction of garlic scape. This anisotropic microstructure directly determines the differences in the material’s mechanical properties along different directions. Drawing on methods from previous studies on the mechanical characterization of plant stems [22–25], the garlic scape is considered as an orthotropic anisotropic material.

**Fig 2:**
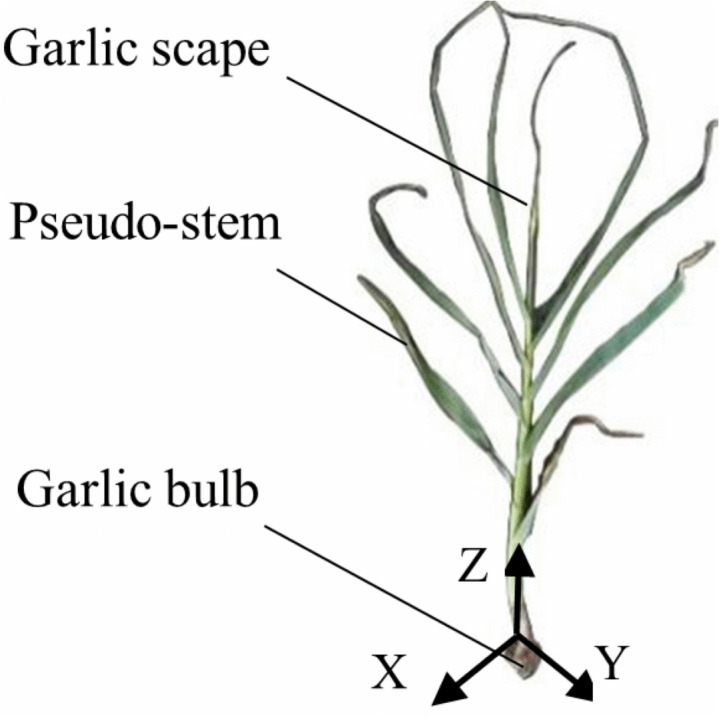
The diagram of a garlic plant.

The garlic plant consists of the garlic bulb, the garlic scape itself and the pseudo-stem that encloses its outer surface. Based on the separation mechanism between the garlic scape and the pseudo-stem during mechanized harvesting, this study focuses on analyzing of the interaction between the pseudo-stem and the garlic scape, so the garlic bulb is not considered. The three-dimensional coordinate axes are established, where the direction along the axis of the garlic plant is defined as the Z-axis, and the directions perpendicular to the axis are the X-axis and Y-axis (as shown in Fig 2).

### 2.5. Mechanical parameters

According to the mechanical properties of composite materials, orthotropic anisotropy materials are characterized by nine parameters, which are axial tensile elastic modulus *E*_*z*_, radial compressive elastic modulus *E*_*x*_ and *E*_*y*_, isotropic plane shear modulus *G*_*xy*_, anisotropic plane shear modulus *G*_*xz*_ and *G*_*yz*_, isotropic plane Poisson ratio *μ*_*xy*_, anisotropic plane Poisson ratio *μ*_*xz*_ and *μ*_*yz*_. According to garlic scape have the characteristics of rotation axis, elastic parameters satisfy formula (3).


$$E_{x}=E_{y}$$

$$u_{xz}=u_{yz}$$

$$G_{xz}=G_{yz}$$

$$G_{xy}=\frac{E_{x}}{2(1+\mu_{xy})}$$ (3)

During the tests, the garlic scape and the pseudo-stem were peeled from the same plant, and the mass of both were weigh by an electronic scale respectively. The respective densities were measured using the drainage method. Based on the measured mass and density data, the volume ratio of the pseudo-stems to the garlic scape was calculated according to Equations (4) and (5).


$$\frac{V_{j}}{V_{t}}=\frac{\frac{m_{j}}{\rho_{j}}}{\frac{m_{t}}{\rho_{t}}}$$ (4)

$$V_{j}+V_{t}=\text{1}$$ (5)

Where *m*_*t*_ and *m*_*j*_ are the mass of garlic scape and the mass of pseudo-stem in the same plant(g), respectively. *ρ*_*t*_ and *ρ*_*j*_ are the density of garlic scape and the density of pseudo-stem (kg/m^3^), respectively. *V*_*t*_ and *V*_*j*_ are the volume ratio of garlic scape and pseudo-stem in the same plant, respectively.

The elastic modulus *E*_*z*_, *E*_*zj*_, *E*_*zt*_ were obtained by axial tensile tests on plants, pseudo-stems and garlic scapes by a universal testing machine, respectively. According to the mixing rule of composite materials [26], elastic modulus is calculated using Equation (6).


$$E_{z}=E_{zj}V_{j}+E_{zt}V_{t}$$ (6)

where *E*_*z*_ is the tensile elastic modulus of garlic plant (MPa). *E*_*zj*_ is the tensile elastic modulus of pseudo-stem (MPa). *E*_*zt*_ is the tensile elastic modulus of garlic scape (MPa). *V*_*t*_ and *V*_*j*_ are the volume ratio of the garlic scape and the pseudo-stem in the same plant, respectively.

Radial compression tests were carried out on plants, pseudo-stems and garlic scapes by a universal testing machine to obtain the compressive elastic modulus *E*_*x*_, *E*_*xj*_ and *E*_*xt*_, respectively. According to the mixing rule of composite materials [26], the following Equation (7) is satisfied.


$$E_{x}=E_{xj}V_{j}+E_{xt}V_{t}$$ (7)

where *E*_*x*_ is the compressive elastic modulus of garlic plant (MPa). *E*_*xj*_ is the compressive elastic modulus of pseudo-stem (MPa). *E*_*xt*_ is the compressive elastic modulus of garlic scape (MPa). *V*_*t*_ and *V*_*j*_ are the volume ratio of the garlic scape and the pseudo-stem in the same plant, respectively.

By conducting shear tests on the plants, pseudo-stems and garlic scapes, the anisotropic plane shear modulus was determined. According to the mixing rule of composite materials [26], the following Equation (8) is satisfied.


$$G_{yz}=\frac{G_{yzj}\times G_{yzt}}{V_{j}G_{yzj}+V_{t}G_{yzt}}$$ (8)

where *G*_*yz*_ is the anisotropic plane shear modulus of garlic plant (MPa). *G*_*yzj*_ is the anisotropic plane shear modulus of pseudo-stem (MPa). *G*_*yzt*_ is the anisotropic plane shear modulus of garlic scape (MPa). *V*_*t*_ and *V*_*j*_ are the volume ratio of the garlic scape and the pseudo-stem in the same plant, respectively.

According to the theory of composite mechanics [26], the anisotropic plane Poisson’s ratio *μ*_*yz*_ can be derived as Equation (9).


$$u_{yz}<\frac{\text{1}}{\sqrt{\text{2}\mu_{xy}}}\times \frac{E_{x}}{E_{z}}$$ (9)

Where *μ*_*yz*_ is the anisotropic plane Poisson’s ratio. *μ*_*xy*_ is the isotropic plane Poisson’s ratio. *E*_*x*_ is the compressive elastic modulus (MPa). *E*_*z*_ is the tensile elastic modulus (MPa).

The shear modulus *G*_*xy*_ could not be directly measured using a universal testing machine and the Poisson’s ratios of similar materials were consulted. It was assumed that the isotropic plane Poisson’s ratio for garlic scape, pseudo-stem, and plant (denoted as *μ*_*xyt*_, *μ*_*xyj*_, and *μ*_*xyz*_, respectively) were all taken to be 0.3 [23], so *G*_*xy*_ could be determined according to Equation (3).

During the implementation of mechanical experiments by the universal testing machine, the stresses are calculated according to Equation (10).


$$\sigma =\frac{F}{A}$$ (10)

where *σ* is stress(N·mm⁻¹). *F* is applied force(N). *A* is cross-sectional area(mm²). The cross-section of the garlic scape and the plant is approximately circular, so


$$\text{A}=\pi {\text{d}}^{\text{2}}/\text{4}$$

where *d* is the cross-sectional diameter(mm).

The cross-section of the pseudo-stem is approximately rectangular, so *A = bh*, where *b* is the width(mm) and *h* is the thickness(mm).

The equation for calculating strain is shown in (11).


$$\varepsilon =\frac{\Delta L}{L}$$ (11)

where *ε* is strain(mm). *∆L* is the longitudinal deformation (mm). *L* is the original longitudinal length before deformation (mm).

### 2.6. Data processing

The mechanical tests provided force-deformation relationships. To obtain mechanical parameters (elastic modulus and ultimate strength) of garlic plants and their components, these relationships were further converted into stress-strain relationships. The calculation formulas for the conversion process were shown in formulas (10) and (11). The elastic modulus was determined by secant modulus, which was calculated according to the slope of the line connecting the origin of stress-strain curve coordinate and the point corresponding to 50% ultimate stress.

In the present study, raw data were first recorded using Microsoft Excel 2020, while Origin 2024 software was employed for statistical analysis and graph plotting.

### 2.7. Mechanical properties

#### 2.7.1. Axial tensile test method.

**Fig 3:**
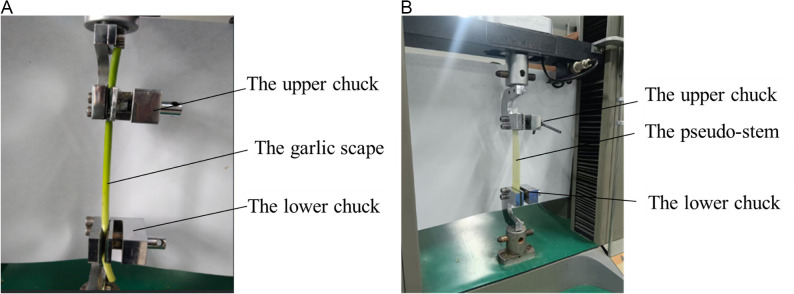
Tensile tests of garlic scapes and pseudo-stems. **(a)** Garlic scape tensile tests. **(b)** Pseudo-stem tensile tests.

In view of the fact that the overall structure of garlic plants is not suitable for axial tensile tests, tensile tests were carried out on garlic scapes and pseudo-stems separately to determine their respective constitutive relationships and mechanical parameters. The tests were conducted by selecting white garlic plants of uniform thickness to make specimens and measuring them. The whole garlic scape was extracted from a plant and samples with a gauge length of 350–500 mm were intercepted to perform axial tensile tests (as shown in Fig 3(a)). To implement pseudo-stem tensile tests, pseudo-stems were made into rectangles with a length of 100 mm, a width of the maximum width of the material itself, and a thickness of the thickness of the material itself (as shown in Fig 3(b)).

In order to prevent slipping during tests, the samples were wrapped with gauze at the clamped part. The universal testing machine carried out tensile tests at the speed of 5 mm/min. The samples were gradually broken with the increase of load, and the tests were successful if the fractures were not at the holding ends. Each test was repeated eight times.

#### 2.7.2. Radial compression test method.

**Fig 4:**
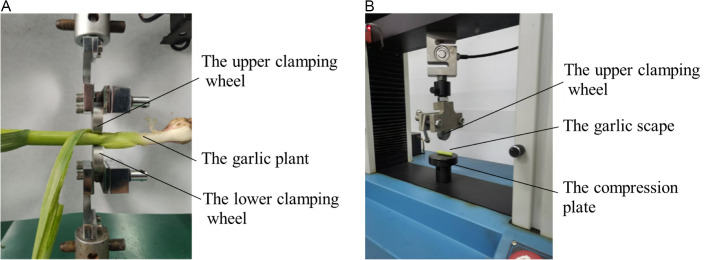
Radial compression tests of garlic plants and garlic scapes. **(a)** Garlic plant compression tests. **(b)** Garlic scape compression tests.

Given the relatively thin pseudo-stem, which was not well-suited for compression testing, radial compression tests were performed on the whole garlic plant and individual garlic scapes. Based on the method of clamping garlic scape [2], and scaled-down model of clamping wheels (thickness of 10 mm, diameter of 50 mm) were installed on the universal testing machine to implement the radial compression tests. The upper chuck of the universal testing machine moved downward with the upper clamping wheel, and the lower clamping wheel was fixed at the lower chuck (Fig 4(a)). The clamped position of garlic plants is 80 mm ~ 100 mm above from the root of garlic plants (consistent with the position of manual clamping). Garlic scapes were prepared by sectioning 20 mm segments along its axial direction, and then placed between the upper clamping wheel and the compression plate of the universal testing machine (Fig 4(b)). The universal testing machine carried out compression tests at the speed of 5 mm/min. Each test was repeated eight times.

#### 2.7.3. Shear test method.

**Fig 5:**
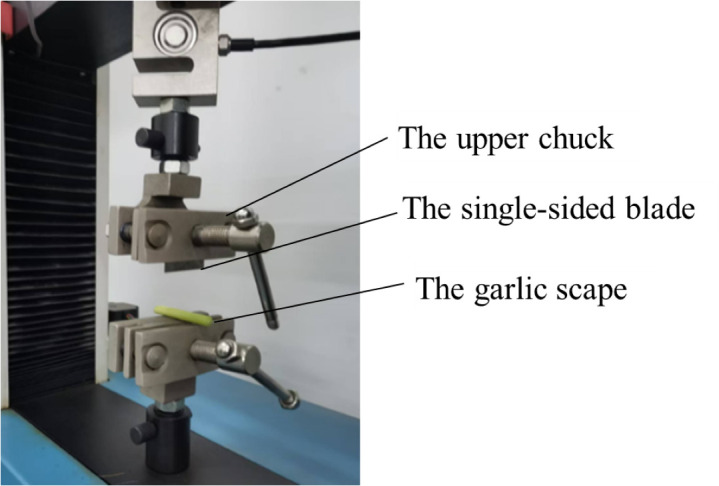
Shear tests of garlic scapes.

Shear tests included plant, garlic scape and pseudo-stem shear tests. The upper chuck on the universal testing machine clamped the single-sided blade and moved downward, as shown in Fig 5.

100mm gauge length sections were cut from plants at 50 mm above the root of garlic plants, and their diameters were measured using a vernier caliper. 50 mm-long samples were cut from the middle part of garlic scape, with their diameters determined using the same measuring instrument. Pseudo-stems were made into rectangles about 30 mm long and 30–40 mm wide along the venation direction, with thicknesses of their own. The loading speed of the universal testing machine is 5 mm/min. Each test was repeated eight times.

### 2.8. Determination of mechanical parameters

Garlic plants exhibit orthotropic anisotropic material properties, and their mechanical behavior is characterized based on the theory of composite material mechanics. Using the average modulus from tensile tests of pseudo-stems and garlic scapes, the tensile modulus of the plants was calculated via formula (6). The average modulus of the eight compression test data of garlic plants and garlic scapes was substituted into formula (7) to obtain the compressive modulus of the pseudo-stems. The isotropic plane Poisson’s ratio and the compressive modulus were substituted into formula (3) to derive the isotropic shear modulus. The anisotropic Poisson’s ratio was then determined by substituting the average value of compressive modulus and tensile modulus into formula (9). Through the above calculations, all mechanical parameters for the garlic plants and their components were obtained.

### 2.9. Finite element modeling and simulation

To verify the reliability and accuracy of the mechanical test results, the finite element method (FEM) was adopted to conduct simulation validation of garlic plant compression tests. The original data from the force-deformation curve measured in the tests was used as the simulation input. The material properties and key parameters of the garlic scapes and pseudo-stems in the simulation model were based on the data determined from aforementioned mechanical tests. Finite element simulation was performed using ANSYS Workbench (Vision:2024R2, ANSYS Inc., USA).

#### 2.9.1. Geometric Model construction.

This study employed SolidWorks (Vision:2018 SP4.0, Dassault, France) for geometric modeling. Geometric models of plants may be simplified according to the research objectives [27,28].To simplify the calculations, the compression section of the garlic scape was modeled as a 20-mm-long solid cylinder. The surrounding multi-layered pseudo-stems were equivalently simplified to a hollow cylinder of uniform thickness. The garlic scape and pseudo-stem parts were assembled using the mating command to construct the complete plant model, with constraints imposed between the two parts to maximize consistency with the actual structural configuration.

#### 2.9.2. Mesh division and contact settings.

**Fig 6:**
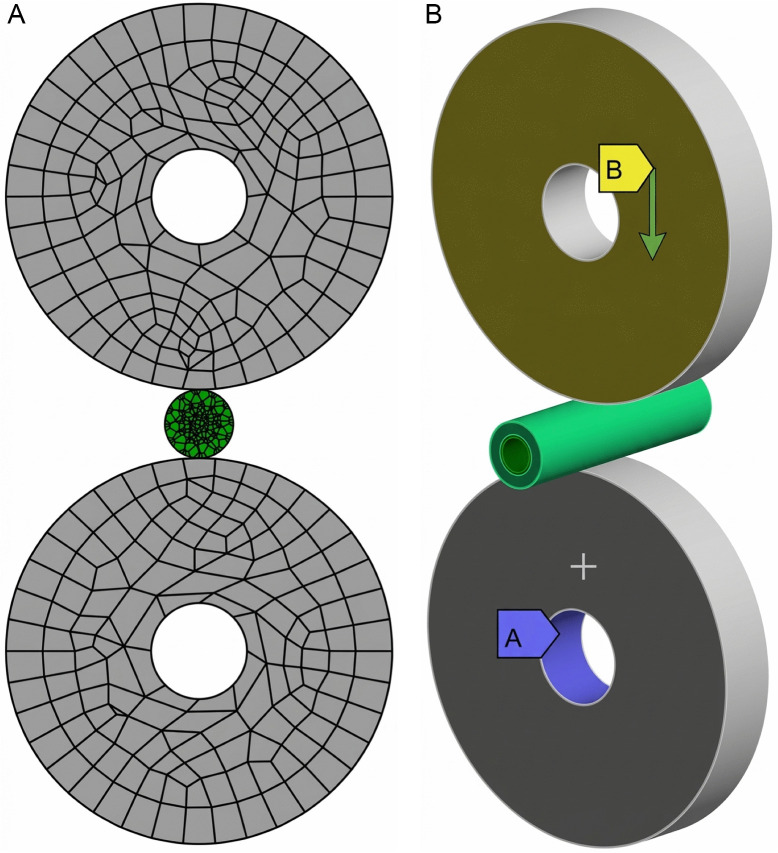
Radial compression ﬁnite element model. **(a)** Grid partitioning. **(b)** Contact settings.

To balance solution accuracy and computational efficiency, a hexahedral mesh was applied to the garlic scape, while a tetrahedral mesh was used for the pseudo-stem. The assembled plant model was imported into the ANSYS/Mesh. A uniform mesh size of 2.5 mm was adopted for both the garlic scape and pseudo-stem, and mesh generation yielded a total of 4982 elements and 13668 nodes. Material parameters for the garlic scape and the pseudo-stem, derived from the mechanical tests conducted in this study, were assigned to their respective models within the simulation. Rigid-flexible contact was defined between the plant model and the clamping wheels. The contact between the pseudo-stem and the garlic scape was defined as a flexible-flexible bonded connection. To prevent lateral slippage during simulation, remote displacement constraints were applied, ensuring that the clamping wheels were restricted to vertical motion only (as shown in Fig 6).

## 3. Results and discussion

### 3.1. Moisture content and density

**Fig 7:**
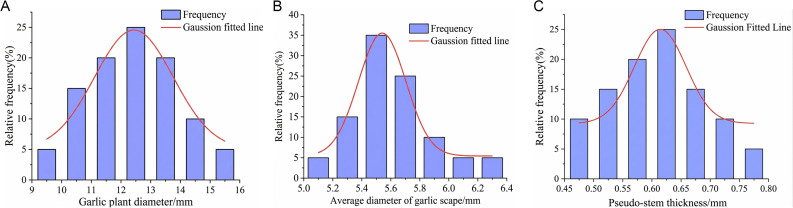
Dimensions of garlic plants and their components. **(a)** Distribution of garlic plant diameter. **(b)** Distribution of the average diameter of garlic scape. **(c)** Distribution of pseudo-stem thickness.

As depicted in Fig 7, the garlic plant diameter, the average diameter of garlic scape and the pseudo-stem thickness conformed to a normal distribution, with median values of 12.44 mm, 5. 59 mm and 0.61 mm, respectively.

The moisture content of fresh garlic scapes was determined to keep 85.88 ± 2.41% by using the 105°C drying method, as illustrated in Table 1.

**Table 1:** Moisture content of garlic scapes.

NO	Fresh weigh(g)	Dry weigh(g)	Moisture content(%)
1	4.215	0.473	88.78%
2	3.065	0.379	87.63%
3	2.143	0.331	84.55%
4	2.895	0.391	86.49%
5	2.547	0.630	85.87%
6	1.956	0.353	81.95%
Average value	2.804	0.426	85.88%
Standard deviation	0.811	0.111	2.41%

The densities of garlic scape and pseudo-stem were measured to be 970 kg/m^3^ and 620 kg/m^3^ respectively by the drainage method. According to the formula (4) and (5), the volume ratio of the garlic scape and the pseudo-stem in the same plant was calculated as follows: *V*_*t*_:*V*_*j*_ = 2:5.

### 3.2. Axial tensile test analysis

**Fig 8:**
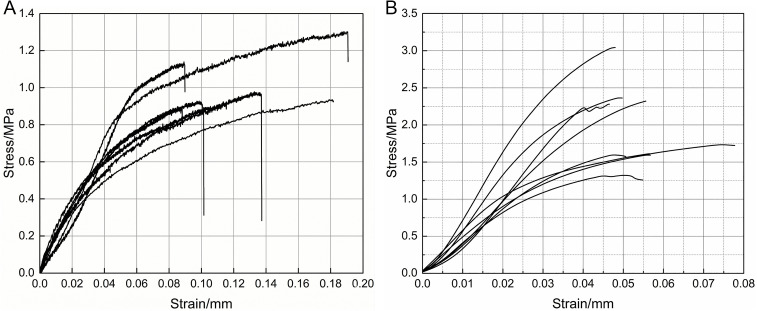
Axial tensile stress-strain curves. **(a)** Axial tensile stress-strain curves of garlic scapes. **(b)** Axial tensile stress-strain curves of pseudo-stems.

Stress-strain curves for the tensile tests of garlic scapes and pseudo-stems are presented in Fig 8, with both exhibiting generally similar trends. In the initial stage of the tests, the tensile stress borne by the garlic scapes and pseudo-stems increases linearly with strain, corresponding to the elastic deformation stage. Beyond this stage, slight inflection points appear in the middle section of the curves, and the growth rate of stress shows a shrinking tendency, entering the plastic deformation stage. When the tensile stress reaches to the maximum, the garlic scapes and pseudo-stems fracture, and the corresponding maximum stress is tensile strength.

The test data are presented in Table 2. The average tensile strength of garlic scapes was 0.98 MPa, the standard deviation was 0.17 MPa, the maximum value was 1.30 MPa, and the minimum value was 0.76 MPa. For the elastic modulus *E*_*zt*_ of garlic scapes, the average value was 17.08 MPa, the standard deviation was 2.67 MPa, the maximum value *E*_*ztmax*_ was 21.55 MPa, and the minimum value *E*_*ztmin*_ was 12.61 MPa. The average peak strain of garlic scapes was 0.12 mm, with a standard deviation of 0.05 mm, the maximum value was 0.19 mm, and the minimum value was 0.05 mm. The average tensile strength of pseudo-stems was 2.04 MPa, with a standard deviation of 0.56 MPa, a maximum value of 3.04 MPa, and a minimum value of 1.32 MPa. For the elastic modulus *E*_*zj*_ of pseudo-stems, the average value was 57.03 MPa, the standard deviation was 12.16 MPa, the maximum value *E*_*zjmax*_ was 81.43 MPa, and the minimum value *E*_*zjmin*_ was 45.04 MPa. The average peak strain of pseudo-stems was 0.06 mm, with a standard deviation of 0.009 mm, the maximum value was 0.07 mm, and the minimum value was 0.05 mm.

**Table 2:** Axial tensile test data.

Material	Diameter (mm)		Length (mm)	Modulus of elasticity (*E*_*zt*_/MPa)	The tensile strength (MPa)	The peak strain (mm)
Garlic scape	5.11		400	17.04	0.93	0.10
	5.36^1^		500	18.20	1.30	0.19
	5.28		365	18.48	0.98	0.13
	4.99		500	15.11	0.92	0.12
	6.39		400	12.61	0.94	0.18
	5.40		390	17.98	0.89	0.09
	5.38		470	15.67	1.14	0.09
	5.25		420	21.55	0.76	0.05
Average Value	5.40		430.63	17.08	0.98	0.12
Standard deviation	0.43		52.27	2.67	0.17	0.05
**Material**	**Thickness** **(mm)**	**Width** **(mm)**	**Length** **(mm)**	**Modulus of Elasticity** **(*E***_***zj***_ **/MPa)**	**The tensile strength (MPa)**	**The peak strain** **(mm)**
Pseudo-stem	0.48	28.0	100	45.04	1.69	0.07
	0.49	24.0	100	46.35	1.32	0.05
	0.39	28.5	100	56.28	2.28	0.05
	0.74	26.0	100	58.56	2.31	0.06
	0.35	22.5	100	46.78	1.59	0.05
	0.31	20.0	100	56.15	1.73	0.07
	0.41	30.0	100	81.43	3.04	0.05
	0.46	28.0	100	65.63	2.36	0.05
Average Value	0.45	25.88	100	57.03	2.04	0.06
Standard deviation	0.13	3.43	0	12.16	0.56	0.009

Variance analysis was performed on the elastic modulus, tensile strength and peak strain of garlic scapes and pseudo-stems using Origin 2024 software at a significance level of *α = 0.05*. The results are presented in Tables 3–5, which reveal that the *p*-values are less than the predetermined significance level (*α = 0.05*). This indicates that there are statistically significant differences in the tensile modulus, tensile strength and peak strain between garlic scapes and pseudo-stems.

**Table 3:** Variance analysis of the tensile modulus of garlic scapes and pseudo-stems.

Sources	Sum of Squares	Degrees of Freedom	Mean Square	F	Significance
Model	6383.211	1	6383.211	82.308	<0.0001
Error	1085.740	14	77.553		
Total	7468.951	15			

**Table 4:** Variance analysis of the tensile strength of garlic scapes and pseudo-stems.

Sources	Sum of Squares	Degrees of Freedom	Mean Square	F	Significance
Model	4.473	1	4.473	26.485	0.00015
Error	2.365	14	0.169		
Total	6.838	15			

**Table 5:** Variance analysis of the peak strain of garlic scapes and pseudo-stems.

Sources	Sum of Squares	Degrees of Freedom	Mean Square	F	Significance
Model	0.01563	1	0.01563	13.44086	0.00254
Error	0.01628	14	0.00116		
Total	0.0319	15			

Based on the tensile test results, the average elastic modulus of garlic scapes is significantly lower than that of the pseudo-stems, amounting to 29.9% of the pseudo-stem value. This indicates that under the same tensile load, garlic scapes are more prone to elastic deformation and exhibit superior elasticity compared to the pseudo-stems. The average tensile strength of garlic scapes is also significantly lower than that of pseudo-stems (the average tensile strength of pseudo-stems being approximately twice that of garlic scapes), indicating that the maximum tensile stress they can withstand is significantly lower than that of pseudo-stems. Meanwhile, the average peak strain of garlic scapes is twice that of pseudo-stems, indicating that the flexibility of garlic scapes is stronger than that of pseudo-stems. This quantitative differences in mechanical properties provide important theoretical support and technical reference for the design of bolting mechanisms and the control of tensile damage during the mechanical harvesting of garlic scapes.

### 3.3. Radial compression test analysis

**Fig 9:**
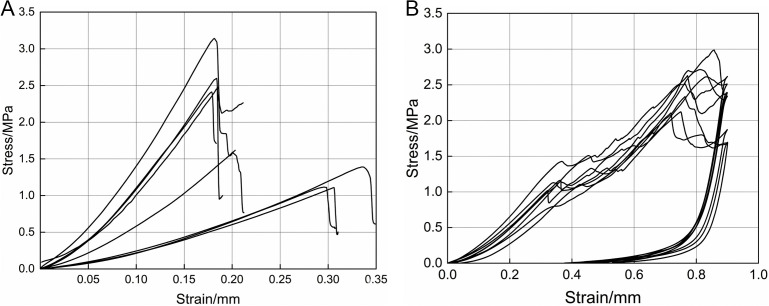
Radial compressive stress-strain curves. **(a)** Radial compressive stress-strain curves of garlic scapes. **(b)** Radial compressive stress-strain curves of garlic plants.

As shown in Fig 9(a), in the stage of compression, the stress increases with the increase in strain and the garlic scape does not exhibit obvious yield characteristics. The stress reaches the peak value, which is the point of compression damage, at which time the compressive strength is in the range of 1.11 to 3.12 MPa.

As shown in Fig 9(b), during the compression process, the garlic plants undergo three stages: non-destructive elastic deformation, bio-yield plastic deformation, and fracture deformation. When the strain is between 0 and 0.4 mm, the plants are in the non-destructive elastic deformation stage, with the stress ranging from 0.8 to 1.5 MPa. Beyond this range, the plants enter the bio-yield plastic deformation stage, where the strain is between 0.4 and 0.6 mm and the stress ranges from 1.0 to 2.0 MPa. Under the continuous action of external loads, the internal tissues of the plants are further damaged, leading to a rapid increase in compressive stress. As stress increases with strain until reaching its peak, the plants occur fracture deformation, during which stress varies within the range of 2.0MPa to 3.0 MPa.

As shown in Table 6, the average compressive strength of garlic scapes was 1.97 MPa with a standard deviation of 0.77 MPa, where the maximum value was 3.12 MPa and the minimum value was 1.11 MPa. For the elastic modulus *E*_*xt*_, the average value was 5.78 MPa with a standard deviation of 2.23 MPa, in which the maximum value *E*_*xtmax*_ was 10.85 MPa and the minimum value *E*_*xtmin*_ was 4.23 MPa. The average peak strain of garlic scapes was 0.22 mm, with a standard deviation of 0.07 mm, the maximum value was 0.34 mm, and the minimum value was 0.18 mm. The average compressive strength of plants was 2.51 MPa with a standard deviation of 0.31 MPa, where the maximum value was 2.99 MPa and the minimum value was 2.08 MPa. For the elastic modulus *E*_*x*_ of plants, the average value was 2.85 MPa with a standard deviation of 0.54 MPa, in which the maximum value *E*_*xmax*_ was 3.95 MPa and the minimum value *E*_*xmin*_ was 2.30 MPa. The average peak strain of garlic plants was 0.80 mm, with a standard deviation of 0.06 mm, the maximum value was 0.90 mm, and the minimum value was 0.72 mm.

**Table 6:** Radial compression test data.

Material	Diameter (mm)	Length (mm)	Modulus of elasticity (*E*_*xt*_/MPa)	The compressive strength (MPa)	The peak strain (mm)
Garlic scape	6.12	20	6.95	1.12	0.18
	6.50	20	4.46	2.39	0.18
	6.43	20	4.25	2.60	0.18
	6.02	20	5.30	3.12	0.18
	5.57	20	4.85	1.57	0.20
	6.41	20	4.23	1.11	0.31
	5.84	20	5.35	1.39	0.34
	5.30	20	10.85	2.48	0.19
Average Value	6.02	20	5.78	1.97	0.22
Standard deviation	0.43	0	2.23	0.77	0.07
**Material**	**Diameter(mm)**		**Modulus of elasticity (*E***_***x***_ **/MPa)**	**The compressive strength (MPa)**	**The peak strain (mm)**
Garlic plant	9.50		3.31	2.62	0.83
	11.82		2.59	2.12	0.75
	11.36		2.57	2.33	0.76
	10.18		2.56	2.70	0.77
	11.14		2.59	2.99	0.86
	9.28		2.89	2.63	0.77
	9.88		3.95	2.62	0.90
	11.73		2.30	2.08	0.72
Average Value Standard deviation	10.61		2.85	2.51	0.80
	1.02		0.54	0.31	0.06

Variance analysis was performed on the compressive modulus, compressive strength and peak strain of garlic scapes and garlic plants at a significance level of *α = 0.05* using Origin 2024 software. The results in Table 7 reveal a *p*-value of *0.0028*, indicating a significant difference in compressive modulus between garlic scapes and garlic plants. Conversely, Table 8 displays a *p*-value of 0.088, suggesting no significant difference in compressive strength between garlic scapes and garlic plants. Table 9 shows a *p*-value less than 0.0001, indicating that the peak strain of garlic scapes differs extremely significantly from that of garlic plants.

**Table 7:** Variance analysis of the compressive modulus of garlic scapes and garlic plants.

Sources	Sum of Squares	Degrees of Freedom	Mean Square	F	Significance
Model	34.457	1	34.457	13.086	0.0028
Error	36.863	14	2.633		
Total	71.320	15			

**Table 8:** Variance analysis of the compressive strength of garlic scapes and garlic plants.

Sources	Sum of Squares	Degrees of Freedom	Mean Square	F	Significance
Model	1.161	1	1.161	3.394	0.088
Error	4.789	14	0.342		
Total	5.950	15			

**Table 9:** Variance analysis of the peak strain of garlic scapes and garlic plants.

Sources	Sum of Squares	Degrees of Freedom	Mean Square	F	Significance
Model	1.3225	1	1.3225	325.96831	<0.0001
Error	0.0568	14	0.00406		
Total	1.3793	15			

In the radial compression tests, there was a significant difference in compressive modulus between garlic scapes and garlic plants, with the compressive modulus of garlic scapes being approximately twice that of the plants. This indicates that under the same external load, garlic scapes exhibit a smaller deformation amplitude, while the plants have better elasticity. The average peak strain of the plants is nearly 3.6 times that of the garlic scapes, demonstrating that garlic scapes are more prone to brittle fracture under small compressive displacement, whereas the plants can withstand large compressive deformation. When the compressive displacement matches the peak strain of the garlic scapes, the garlic scapes undergo brittle fracture; At this time, the plants are in the elastic deformation stage or the transition stage between elastic and bio-yield plastic deformation. This study clarifies the differences in compressive mechanical properties between garlic scapes and garlic plants, providing a theoretical basis for realizing garlic scape-garlic plant separation in garlic scape harvesters based on clamping principle.

### 3.4. Shear test analysis

**Fig 10:**
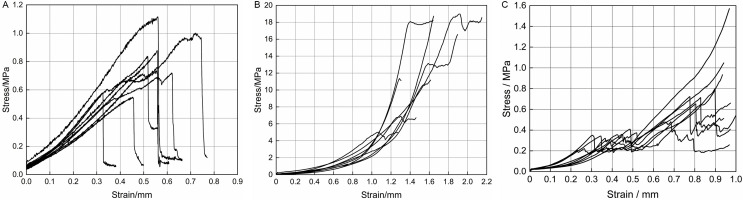
Shear stress-strain curves. **(a)** Shear stress-strain curves of garlic scapes. **(b)** Shear stress-strain curves of pseudo-stems. **(c)** Shear stress-strain curves of garlic plants.

As shown in Fig 10(a), the stress of garlic scapes shows an overall increasing trend with the strain. When the strain is in the range of 0–0.25 mm, the garlic scapes are in the elastic deformation stage, with the shear stress ranging from 0.25 MPa to 0.6 MPa. When the shear stress increases to the maximum value, the garlic scapes fracture and the bearing capacity decreases greatly. At this point, the strain is in the range of 0.35–0.8 mm, and the shear strength ranges from 0.54 MPa to 1.12 MPa.

As shown in Fig 10(b), during the initial stage, the pseudo-stems exhibit elastic deformation within the strain range of 0–0.6 mm, with the shear stress ranging from 0.67–1.59 MPa. Subsequently, the stress displays a nonlinear increase with strain until reaching the peak value. At this time, the shear strength is measured between 3.45 MPa and 18.98 MPa.

As shown in Fig 10(c), during the shearing process of plants, the stress increases linearly with strain in the elastic deformation stage, and the yield characteristic is obvious. As strain continues to increases, the plants enter the plastic deformation stage, during which stress rises sharply until reaching its maximum value. At this point, the plants fracture, with shear strength ranging from 0.48 to 1.58 MPa.

As shown in Table 10, the statistical results of shearing tests indicate that the average shear strength of garlic scapes was 0.80 MPa with a standard deviation of 0.20 MPa, where the maximum value was 1.12 MPa and the minimum value was 0.54 MPa. For the elastic modulus *G*_*yzt*_ of garlic scapes, the average value was 1.53 MPa with a standard deviation of 0.26 MPa, in which the maximum value was 2.01 MPa and the minimum value was 1.15 MPa. The average peak strain of garlic scapes was 0.54 mm, with a standard deviation of 0.12 mm, the maximum value was 0.72 mm, and the minimum value was 0.33 mm. For pseudo-stems, the average shear strength was 12.48 MPa with a standard deviation of 6.02 MPa, the maximum value was 18.98 MPa, and the minimum value was 3.45 MPa. The average value of the shear modulus *G*_*yzj*_ of pseudo-stems was 5.21 MPa with a standard deviation of 1.50 MPa, the maximum value was 7.45 MPa and the minimum value was 3.35 MPa. The average peak strain of pseudo-stems was 1.48 mm, with a standard deviation of 0.26 mm, the maximum value was 1.92 mm, and the minimum value was 1.07 mm. The average shear strength of plants was 0.87 MPa with a standard deviation of 0.34 MPa, a maximum value of 1.58 MPa, and a minimum value of 0.48 MPa. For the elastic modulus *G*_*yz*_ of garlic plants, the average elastic modulus was 1.45 MPa with a standard deviation of 0.23 MPa, a maximum value of 1.99 MPa, and a minimum value of 1.26 MPa. The average peak strain of garlic plants was 0.85 mm, with a standard deviation of 0.10 mm, the maximum value was 0.97 mm, and the minimum value was 0.68 mm.

**Table 10:** Shear test data.

Material	Diameter (mm)		Length (mm)	Modulus of elasticity (*G*_*yzt*_/MPa)	The shear strength (MPa)	The peak strain (mm)
Garlic scape	6.02		50	1.36	0.99	0.72
	6.60		50	1.15	0.54	0.45
	5.90		50	1.56	0.87	0.56
	6.88		50	1.72	0.84	0.52
	5.98		50	1.42	0.73	0.56
	6.88		50	1.58	0.58	0.33
	5.18		50	2.01	1.12	0.56
	7.68		50	1.42	0.72	0.62
Average Value	6.39		50	1.53	0.80	0.54
Standard deviation	0.77		0	0.26	0.20	0.12
**Material**	**Thickness** **(mm)**	**Width** **(mm)**	**Length** **(mm)**	**Modulus of elasticity** **(*G***_***yzj***_ **/MPa)**	**The shear strength (MPa)**	**The peak strain (mm)**
Pseudo-stem	0.78	34	30	3.35	5.03	1.07
	0.68	32	30	3.44	3.45	1.30
	0.52	38	30	5.03	13.13	1.59
	0.68	32	30	6.45	18.98	1.92
	0.70	36	30	5.14	11.32	1.29
	0.78	39	30	4.25	11.14	1.61
	0.6	38	30	7.45	18.04	1.41
	0.6	38	30	6.53	18.77	1.64
Average Value Standard deviation	0.67	35.88	30	5.21	12.48	1.48
	0.09	2.85	0	1.50	6.02	0.26
**Material**	**Diameter** **(mm)**	**Length** **(mm)**	**Modulus of elasticity** **(*G***_***yz***_ **/MPa)**		**The shear strength** **(MPa)**	**The peak strain** **(mm)**
Garlic plant	10.60	100	1.42		1.05	0.94
	10.30	100	1.32		0.48	0.68
	13.84	100	1.36		0.78	0.78
	10.60	100	1.49		0.65	0.80
	10.35	100	1.99		1.58	0.97
	11.30	100	1.46		0.79	0.89
	12.00	100	1.33		0.69	0.82
	10.66	100	1.26		0.94	0.93
Average Value Standard deviation	11.21	100	1.45		0.87	0.85
	1.20	0	0.23		0.34	0.10

Variance analysis was performed on the shear modulus, shear strength and peak strain of garlic scapes and pseudo-stems using Origin 2024 software at a significance level of *α = 0.05*. The results are presented in Tables 11–13, which reveal that the *p*-values are less than *0.0001*. This indicates that there are statistically significant differences in the shear modulus, shear strength and peak strain between garlic scapes and pseudo-stems.

**Table 11:** Variance analysis of the shear modulus of garlic scapes and pseudo-stems.

Sources	Sum of Squares	Degrees of Freedom	Mean Square	F	Significance
Model	54.096	1	54.096	46.417	<0.0001
Error	16.316	14	1.165		
Total	70.412	15			

**Table 12:** Variance analysis of the shear strength of garlic scapes and pseudo-stems.

Sources	Sum of Squares	Degrees of Freedom	Mean Square	F	Significance
Model	546.040	1	546.040	30.143	<0.0001
Error	253.607	14	18.115		
Total	799.647	15			

**Table 13:** Variance analysis of the peak strain of garlic scapes and pseudo-stems.

Sources	Sum of Squares	Degrees of Freedom	Mean Square	F	Significance
Model	3.52501	1	3.52501	84.7521	<0.0001
Error	0.58229	14	0.04159		
Total	4.10729	15			

In the shear test, the average shear modulus of pseudo-stems is approximately 3.4 times that of garlic scapes, indicating that under the same shear load, pseudo-stems exhibit a smaller shear deformation amplitude and higher stiffness, while garlic scapes have lower shear stiffness and greater deformation adaptability. The average shear strength of pseudo-stems is significantly higher than that of garlic scapes (15.6 times greater), demonstrating that pseudo-stems have stronger resistance to shear failure and are less prone to shear fracture. Additionally, the peak strain of pseudo-stems is larger than that of garlic scapes, suggesting that garlic scapes are prone to fracture failure under small shear displacement, whereas pseudo-stems can tolerate a greater degree of shear deformation. When the shear displacement matches the peak strain of garlic scapes, garlic scapes undergo shear fracture. At this point, pseudo-stems remain in the elastic deformation stage or the transition stage between elastic and plastic deformation, with no irreversible structural damage occurring. This study clarifies the differences in shear mechanical properties between garlic scapes and pseudo-stems, providing a theoretical basis for the parameter optimization of shear mechanisms in garlic scape harvesters.

### 3.5. Calculation of mechanical parameters

The tensile modulus of garlic plants *E*_*z*_ is calculated by substituting *E*_*zj*_ and *E*_*zt*_ into Equation (6), and the result is 45.62 MPa. The compressive modulus of pseudo-stems *E*_*xj*_ is calculated by substituting *E*_*x*_ and *E*_*xt*_ into Equation (7), and the result is 1.68 MPa.

The shear modulus of the isotropic plane is calculated by bringing *E*_*x*_, *E*_*xt*_, *E*_*xj*_ and *μ*_*xyz*_, *μ*_*xyt*_, *μ*_*xyj*_ into Equation (3). The calculated results are as follows: the shear modulus *G*_*xy*_ of garlic plants is 1.10 MPa, the shear modulus *G*_*xyt*_ of garlic scapes is 2.22 MPa, and the shear modulus *G*_*xyj*_ of pseudo-stems is 0.66 MPa.

By substituting the values of *E*_*x*_ and *E*_*z*_ into Equation (9), the calculated results show that the Poisson’s ratio *μ*_*yz*_ of garlic scapes is less than 0.44, that of pseudo-stems is less than 0.04, and that of whole plants is less than 0.08.

Based on the above experimental data and calculation results, elastic parameters of garlic plants and their components are presented in Table 14.

**Table 14:** Elasticity parameters of garlic plants and components.

Material	*E*_*x*_	*E*_*y*_	*E*_*z*_	*G*_*xy*_	*G*_*xz*_	*G*_*yz*_	*μ*_*xy*_	*μ*_*xz*_	*μ*_*yz*_
Garlic scape	5.78	5.78	17.08	2.22	1.53	1.53	0.3	<0.44	<0.44
Pseudo-stem	1.68	1.68	5 7.03	0.66	5.21	5.21	0.3	<0.04	<0.04
Garlic plant	2.85	2.85	45.62	1.10	1.45	1.45	0.3	<0.08	<0.08

### 3.6. Finite element simulation results

**Fig 11:**
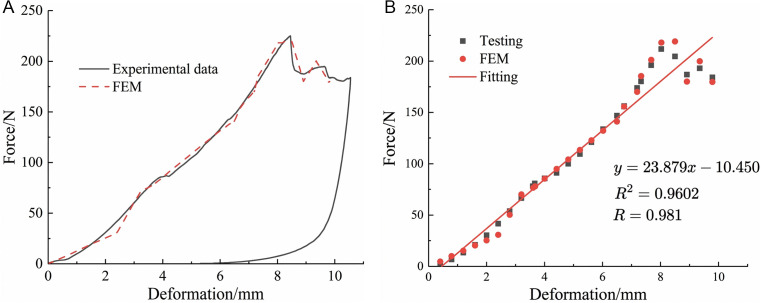
Comparison of simulation and experimental results. **(a)**Simulation and experimental results of compression test of garlic plant. **(b)** Regression analysis of compression test results and simulation results.

As shown in Fig 11, by comparing the radial compression force-deformation curves obtained from simulation and experiments, it can be seen that the two are in good overall agreement and show a significant positive correlation (*R = 0.981*). However, during the plastic deformation stage, the curve shows a slight deviation, which may be due to the certain limitations of the finite element model in simulating the cohesion between pseudo-stems and garlic scapes. To further verify the key mechanical parameters (elastic modulus) of the model in the elastic stage, we conducted a fitting analysis on the data of this stage. The regression equation has a relatively high degree of fit (*R² = 0.9602*), which confirms that the established finite element model can accurately characterize the mechanical response characteristics of the plant during the elastic deformation stage, thereby verifying the reliability of the tensile and compression test results.

### 3.7. Verification of the shear tests

Further examination of the test results is necessary to validate the accuracy of the mechanics experiments. Due to the unique growth characteristics of garlic plants, tensile tests were not performed. Similarly, compression tests were also not carried out because of the thin pseudo-stem thickness. Based on the mixing rule of composite materials, only the parameters of the plant shear tests were validated.

**Fig 12:**
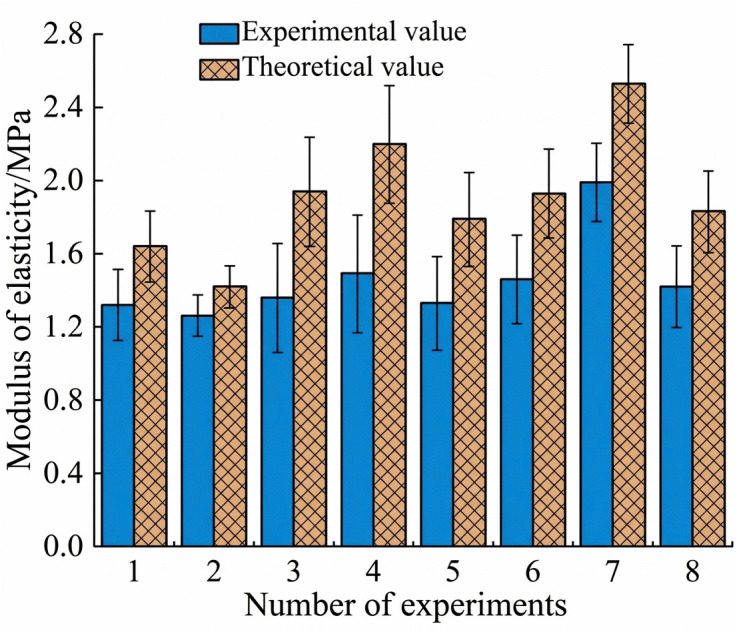
Comparison of theoretical and experimental values for shear tests.

In the shear tests, the theoretical shear modulus of garlic plants is calculated as 1.92 MPa by substituting the shear modulus of garlic scapes and that of pseudo-stems into Equation (8). However, the average shear modulus of plants measured in the tests is 1.45 MPa, which falls outside the 95% confidence interval [1.26, 1.65] MPa of the actual tests, with a relative deviation of 25.5% compared to the tests. As shown in Fig 12, in the shear tests, the theoretically calculated values of the shear modulus are all greater than the experimental values.

**Fig 13:**
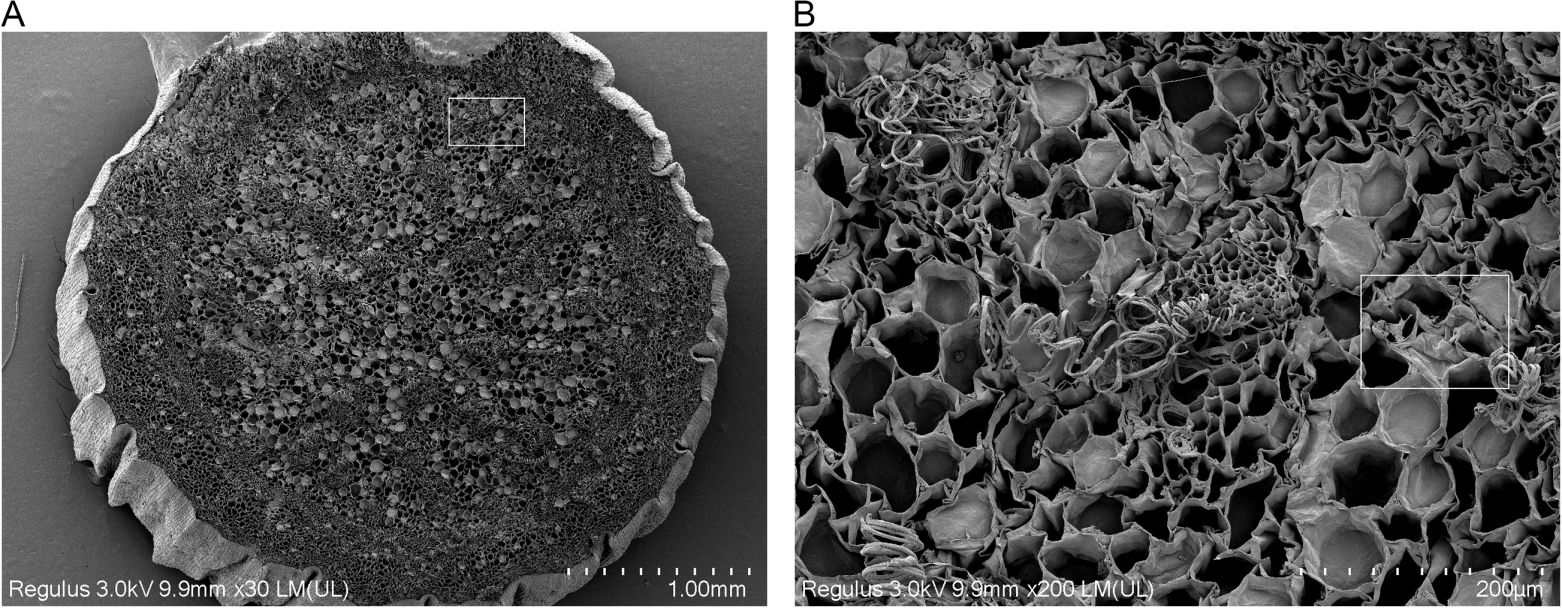
SEM image of the shear transverse section of garlic scapes. **(a)** Overall morphology at a magnification of 30 × . **(b)** Observed cell collapse at 200 × magnification.

**Fig 14:**
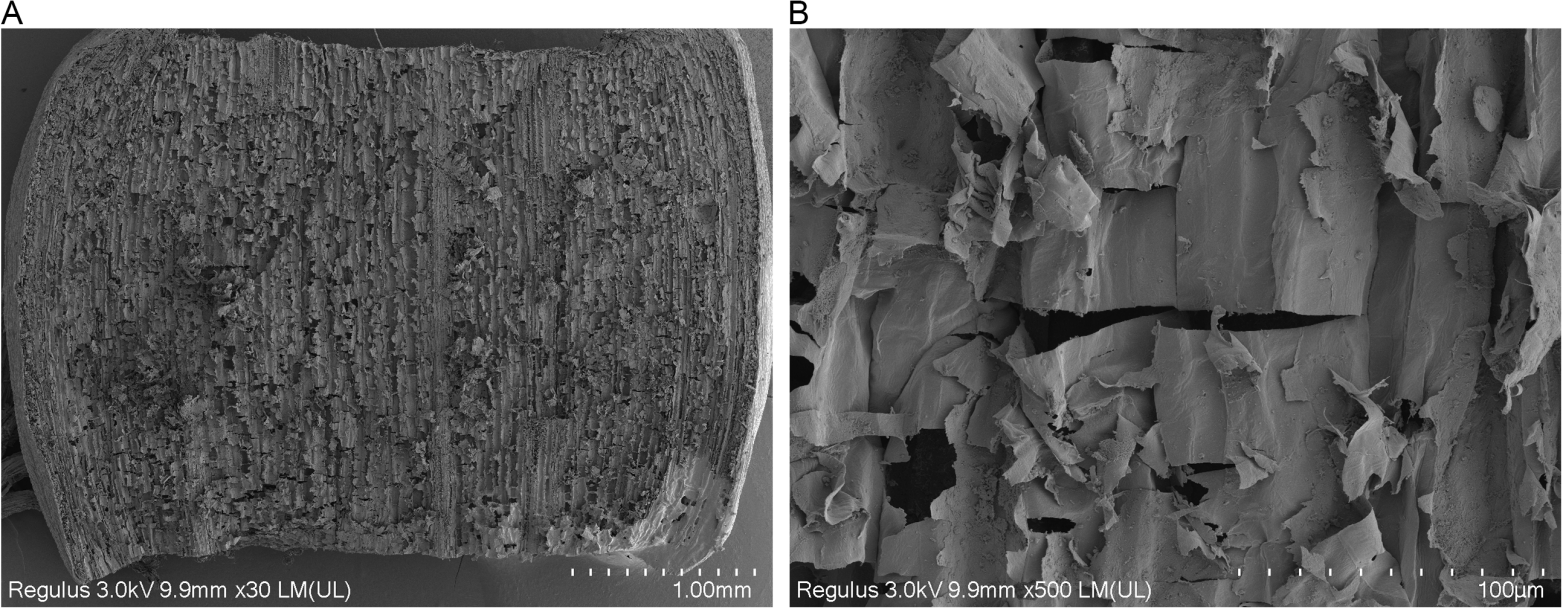
SEM image of the shear longitudinal section of garlic scapes. **(a)** Overall morphology at a magnification of 30 × . **(b)** Observed cell damage at 200 × magnification.

To reveal the microscopic mechanism of this mechanical property difference, SEM observations were conducted on garlic scape tissues. Fig 13 shows the SEM morphology of the transverse section of garlic scapes after shear testing. Compared with the transverse section of the garlic scape samples without the shear test (Fig 1(a)), a large number of originally plump, round parenchyma cells exhibit significant collapse. Fig 14 presents the SEM morphology of the longitudinal section of garlic scapes after shearing testing. In contrast to the longitudinal section of intact garlic scape samples (Fig 1(b)), the cell layers in the sheared longitudinal section exhibit obvious fractures, local stacking, and wrinkling phenomena. Through comparative analysis, these microstructural changes indicate that cell slippage occurred within the garlic scape tissue during the shear process.

### 3.8. Discussion

In this study, we systematically investigated the physical and mechanical properties of garlic plants and their components. The results reveal the differences in mechanical responses between garlic scapes and pseudo-stems under various loading modes, providing a key scientific basis for achieving selective separation of “garlic scape breaking without pseudo-stem damage” in mechanized harvesting.

During axial tensile loading, significant differences (*p < 0.05*) are observed in the elastic modulus, tensile strength, and peak strain between garlic scapes and pseudo-stems, which are primarily determined by their distinct biological tissue structures. Garlic scapes have a larger transverse-sectional size than pseudo-stems, are rich in parenchyma cells, and have a relatively loose texture. In contrast, pseudo-stems are thinner in thickness, with extremely dense cell arrangement and a higher degree of fibrosis. Under external loading, pseudo-stems exhibit less deformation due to their dense structure, thereby demonstrating higher mechanical strength. The test results show that during the mechanical harvesting of garlic scapes, by adjusting the structural parameters of the bolting device, the bolting stress is controlled within the range of 0.98MPa to ensure that garlic scapes do not break. This approach lays the foundation for the breakage rate of garlic scapes.

**Fig 15:**
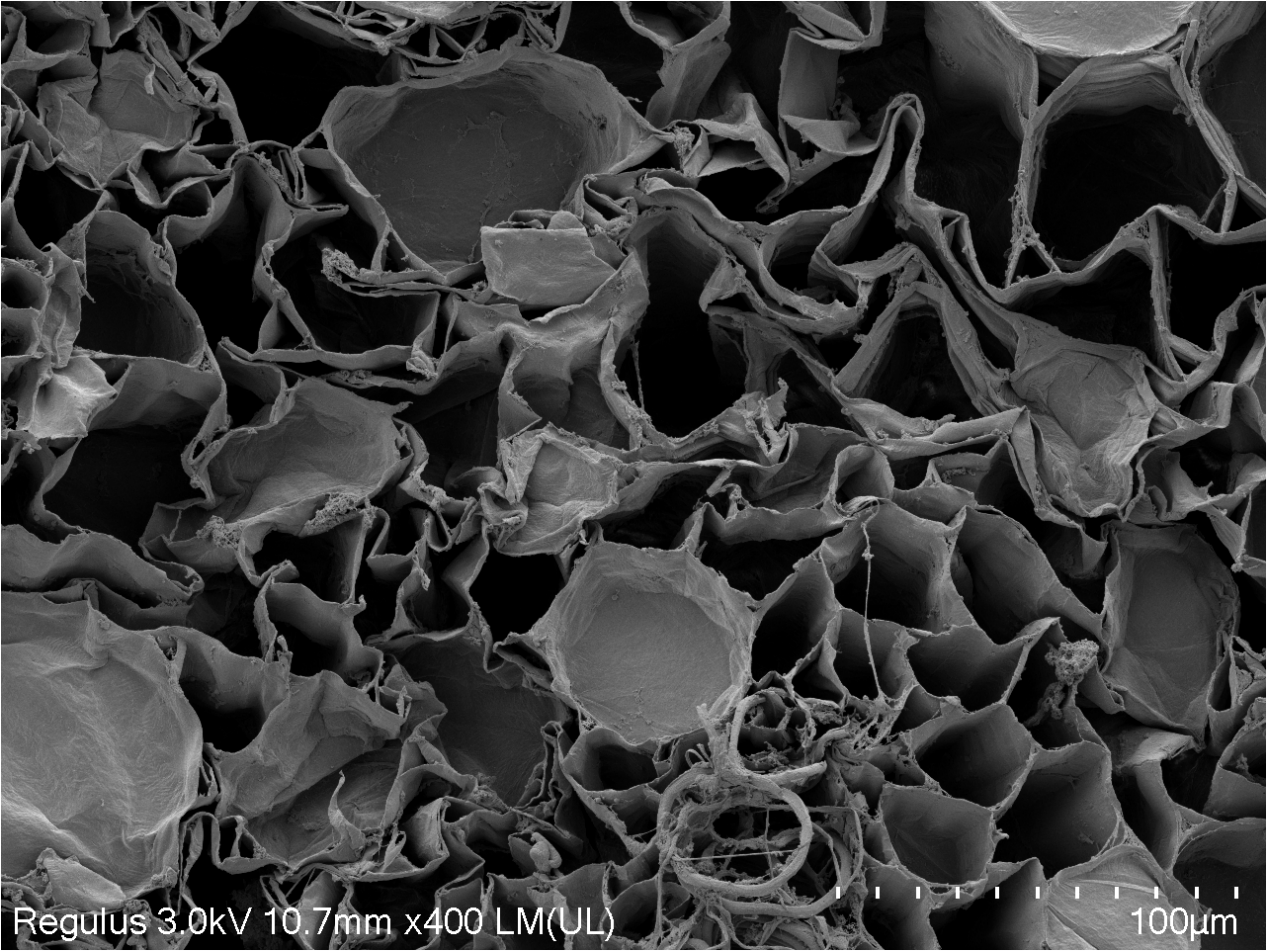
SEM image of the compression transverse section of garlic scapes at 400 × magnification.

Under radial compression, the elastic modulus of garlic scape is significantly higher than that of garlic plant (*p < 0.05*). This indicates that during the initial stage of compression, the cells of garlic scape collapse and the intercellular spaces are rapidly compressed (Fig 15), resulting in a higher elastic modulus in this phase.

In contrast, during the initial compression of the whole garlic plant, the process primarily involves the compaction of gaps between the multiple layers of pseudo-stems that envelop the garlic scape. This stage requires relatively lower loading force, after which the deformation of the pseudo-stem and the garlic scape itself begins. Consequently, the overall deformation is greater, and the elastic modulus is relatively lower. The significant difference in peak strain (*p < 0.05*) between the garlic scape and the whole garlic plant provides a key parameter for selective harvesting based on the principle of clamping garlic scape. By adjusting the gap between the two clamping wheels to control the compressive strain of garlic plant within the range of 0.22–0.80 mm, the selective harvesting objective of “breaking the garlic scape while keeping the pseudo-stem intact” can be achieved.

Under radial shear loading, the elastic modulus, shear strength, and peak strain of garlic scapes are significantly lower than those of pseudo-stems (*p < 0.05*). This indicates that garlic scapes exhibit greater brittleness and weaker shear resistance under shear loads. The underlying mechanism lies in the fact that during the shearing process, the parenchyma cells inside garlic scapes are prone to stacking, compression, and rapid rupture. In contrast, due to higher cellulose content, pseudo-stems can better resist shear deformation and display greater toughness.

Shear modulus were validated based on the theory of composite materials mechanics, showing that the theoretical values were higher than the experimental results. This discrepancy is mainly attributed to the occurrence of microscopic slippage, interface separation, and uneven stress transmission when the shear stress borne between cells exceeds their bonding strength under shear loading. These phenomena reduce the overall structural stiffness, which consequently leads to the theoretical values using the mixing rule of composite materials to be higher than the experimental values.

In summary, the garlic plant is a composite structure consisting of multiple layers of pseudo-stems encasing garlic scapes. Under radial loading (compression or shearing), the garlic scapes exhibit significant deformation or failure with relatively small displacements. This conclusion is consistent with the previous findings on radial compression damage of fruits [11–20]. For instance, Pu et al. [12] reported that in navel oranges compressed by 10 mm, only minor tearing appeared on the peel surface, while the pulp had exceeded its yield stress, resulting in irreversible damage. Cheng et al. [13] found that during the compression of Cerasus humilis, although the peel remained intact, the pulp had already entered the plastic deformation stage, indicating internal damage. Li et al. [17] concluded that when kiwifruit was subjected to a pressure of 25 N, the magnitude of the strain change in the pulp was significantly higher than that of the peel. Similarly, for grapefruits under longitudinal compression, the peel reached its yield limit at a deformation of 29.47 mm, whereas the pulp tissue had already yielded at a compression displacement of 10 mm [11].

It is worth noting that the characteristic of “inner layer damage preceding external failure” in fruits has primarily been applied to guide damage control during harvesting and transportation. In contrast, the analogous behavior observed between garlic scapes and pseudo-stems provides a critical scientific basis for achieving selective separation during mechanized harvesting. This approach helps reduce the rate of garlic scape breakage and improve operational quality.

## 4. Conclusions

The core of mechanized harvesting of garlic scapes is to achieve selective separation of garlic scapes from pseudo-stems. In this study, systematic mechanical tests were conducted on garlic plants and their components (garlic scapes and pseudo-stems), and the differences in mechanical properties between garlic scapes and pseudo-stems were thoroughly analyzed and verified. This provides critical theoretical support and data basis for mechanized selective harvesting. The main results are as follows:

(1) The garlic plants are composite material with multi-scale structures, and scanning electron microscopy (SEM) characterization confirmed their orthotropic anisotropic characteristics. Key physical parameters were measured: the diameter of garlic plants, the diameter of garlic scapes, and the thickness of pseudo-stems all followed a normal distribution, with mean values of 12.44 mm, 5.59 mm, and 0.61 mm, respectively. The average moisture content of fresh garlic scapes was 85.88%. The densities of garlic scapes and pseudo-stems are 970 kg/m³ and 620 kg/m³, respectively.
(2) Through axial tensile, radial compressive, and radial shear tests, the core mechanical parameters (elastic modulus, ultimate strength, peak stress) of garlic plants, garlic scapes, and pseudo-stems were systematically obtained. Statistical analysis results revealed that there were significant differences in the aforementioned mechanical parameters between garlic scapes and pseudo-stems, providing a mechanical basis for their selective separation.
(3) Finite element simulation was used to verify the results of compression tests. The simulation curve showed a high degree of agreement with the test curve, confirming the reliability of the compression test and tensile test data. Based on the theory of composite material mechanics, the results of garlic plant shear tests were analyzed, and it was found that the theoretical values of the garlic plant’s shear modulus were all higher than the measured values. Combined with SEM image observation, it indicates that when the shear stress borne between cells exceeds their bonding strength, microscopic slippage, interface separation and uneven stress transmission occur, which is the main reason for the above differences.
(4) The comparison results of the mechanical properties of garlic scapes and pseudo-stems showed that when garlic plants are subjected to radial loading, garlic scapes are damaged at a smaller deformation, and their fracture behavior occurs earlier than that of pseudo-stems. This characteristic provides direct data support for achieving the selective separation of “garlic scape breakage without pseudo-stem damage” during mechanized harvesting.

Existing studies have not adequately considered the influence of moisture content on the mechanical properties of garlic plants and their components, which may limit the validity of experimental conclusions in practical applications. Meanwhile, the garlic plant is a natural multiscale composite material. The heterogeneity of its microstructure results in the effective stiffness of the plant at the macroscopic scale being lower than the theoretical predictions based on the assumption of an **“**ideal homogeneous continuum**”**. Therefore, when applying the mixing rule of composite materials for mechanical modeling, it is essential to fully consider the heterogeneity of plant tissues at the cellular and tissue levels, as well as the biological specificity of interfacial bonding, to more accurately reflect their mechanical responses.

Future work will conduct more systematic mechanical tests under the condition of considering moisture content and further develop constitutive models that can reflect the heterogeneity and structural non-ideality of garlic plants, thereby further enhancing the understanding and predictive capability of the separation mechanism between garlic scapes and pseudo-stems.

## Supporting information

S1 Fig Garlic scape planting base. The field growth state of garlic plants during the harvest period.
(TIF)

S2 Fig Picked experimental samples. (TIF)

S3 Fig Measurement of diameters and lengths. (TIF)

S4 Fig Weighing of garlic scapes. (TIF)

S5 Fig Weighing of pseudo-stems. (TIF)

S6 Fig Garlic scape samples for tensile tests. (TIF)

S7 Fig Process of garlic scape tensile tests. (TIF)

S8 Fig Process of pseudo-stem tensile tests. (TIF)

S9 Fig Samples of compression tests. (TIF)

S10 Fig The compression tests of garlic plants. (TIF)

S11 Fig The compression tests of garlic scapes. (TIF)

S12 Fig Garlic scape samples for shear tests. (TIF)

S13 Fig Pseudo-stem samples for shear tests. (TIF)

S14 Fig Process of garlic scape shear tests. (TIF)

S15 Fig Garlic scape shearing effect diagram. (TIF)

S16 Fig Pseudo-stem shearing effect diagram. (TIF)
