# Supplementary figures and images for: Experimental studies on the mechanical properties of garlic scape
Source: PLoS One. 2026 Apr 16;21(4):e0344722. doi: 10.1371/journal.pone.0344722 (PMC13086434; doi:10.1371/journal.pone.0344722)

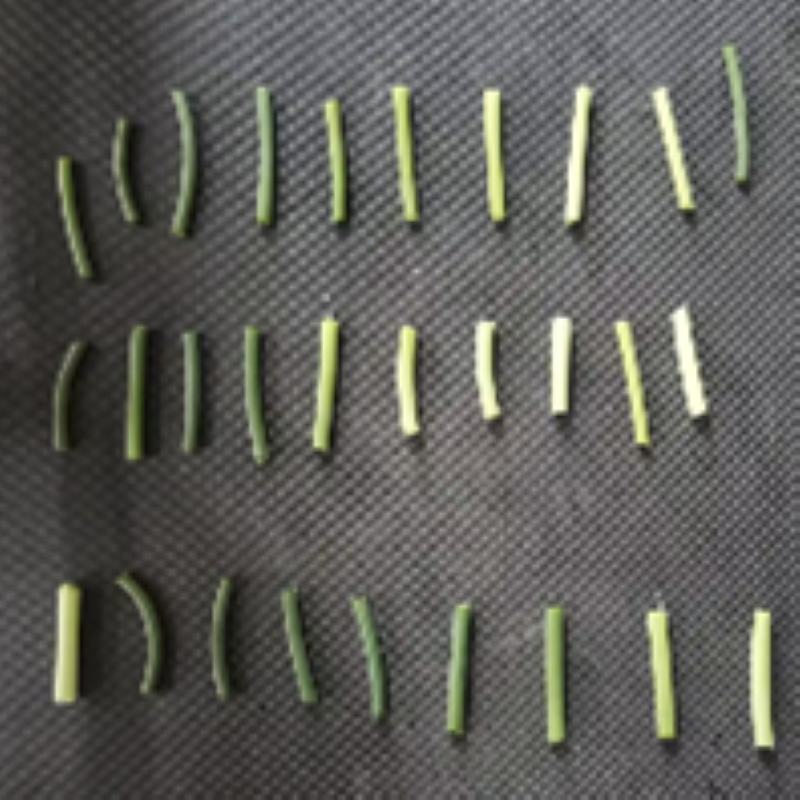

Supplement: S12 Fig — (TIF) [file pone.0344722.s012.tif]

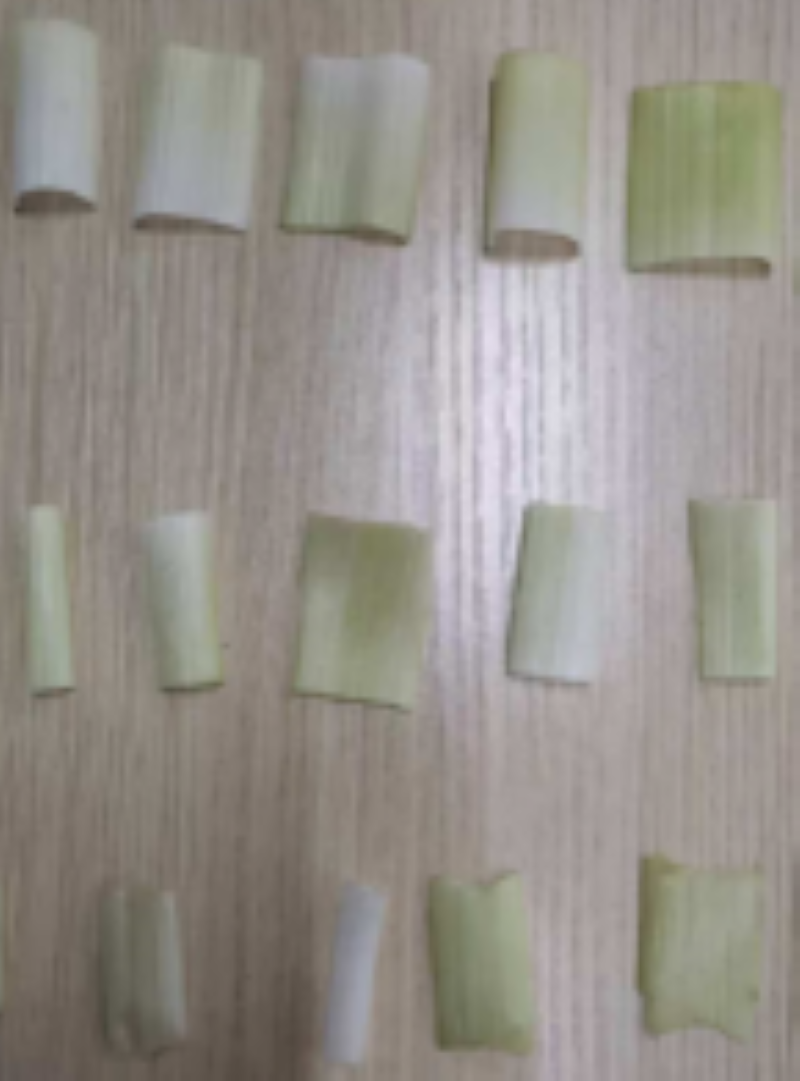

Supplement: S13 Fig — (TIF) [file pone.0344722.s013.tif]

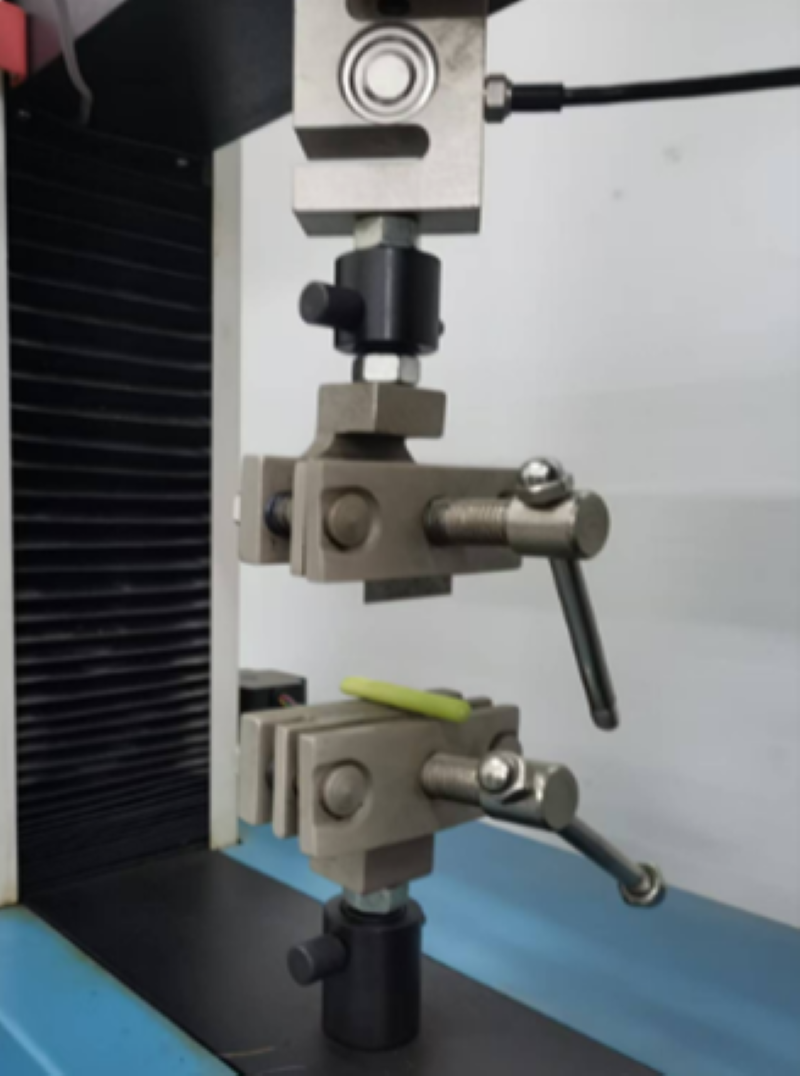

Supplement: S14 Fig — (TIF) [file pone.0344722.s014.tif]
